# Induced pluripotent stem cells as natural biofactories for exosomes carrying miR-199b-5p in the treatment of spinal cord injury

**DOI:** 10.3389/fphar.2022.1078761

**Published:** 2023-01-10

**Authors:** Jun Li, Yingli Jing, Fan Bai, Ying Wu, Limiao Wang, Yitong Yan, Yunxiao Jia, Yan Yu, Benzhi Jia, Fawad Ali

**Affiliations:** ^1^ Department of Spinal and Neural Functional Reconstruction, China Rehabilitation Research Center, Beijing, China; ^2^ School of Rehabilitation Medicine, Capital Medical University, Beijing, China; ^3^ China Rehabilitation Science Institute, Beijing, China; ^4^ State Key Laboratory of Proteomics, National Center for Protein Sciences (Beijing), Beijing Institute of Lifeomics, Beijing, China; ^5^ College of Rehabilitation Medicine, Shandong University of Traditional Chinese Medicine, Jinan, Shandong, China; ^6^ Department of Spinal cord injury rehabilitation, Shanxi Kangfu Hospital, Xi’an, Shanxi, China; ^7^ Department of Pharmacy, Kohat University of Science and Technology, Kohat, Pakistan

**Keywords:** induced pluripotent stem cells-derived exosomes, spinal cord injury, microphage polarization, miR-199b-5p, Hgf, quantitative RT-PCR assay

## Abstract

**Background:** Induced pluripotent stem cells-derived exosomes (iPSCs-Exo) can effectively treat spinal cord injury (SCI) in mice. But the role of iPSCs-Exo in SCI mice and its molecular mechanisms remain unclear. This research intended to study the effects and molecular mechanism of iPSCs-Exo in SCI mice models.

**Methods:** The feature of iPSCs-Exo was determined by transmission electron microscope (TEM), nanoparticle tracking analysis (NTA), and western blot. The effects of iPSCs-Exo in the SCI mice model were evaluated by Basso Mouse Scale (BMS) scores and H&E staining. The roles of iPSCs-Exo and miR-199b-5p in LPS-treated BMDM were verified by immunofluorescence, RT-qPCR, and Cytokine assays. The target genes of miR-199b-5p were identified, and the function of miR-199b-5p and its target genes on LPS-treated BMDM was explored by recuse experiment.

**Results:** iPSCs-Exo improved motor function in SCI mice model *in vivo*, shifted the polarization from M1 macrophage to M2 phenotype, and regulated related inflammatory factors expression to accelerate the SCI recovery in LPS-treated BMDM *in vitro*. Meanwhile, miR-199b-5p was a functional player of iPSCs-Exo, which could target hepatocyte growth factor (Hgf). Moreover, miR-199b-5p overexpression polarized M1 macrophage into M2 phenotype and promoted neural regeneration in SCI. The rescue experiments confirmed that miR-199b-5p induced macrophage polarization and SCI recovery by regulating Hgf and Phosphoinositide 3-kinase (PI3K) signaling pathways.

**Conclusion:** The miR-199b-5p-bearing iPSCs-Exo might become an effective method to treat SCI.

## Introduction

Spinal cord injury (SCI) can result in permanent motor and sensory deficits (Anjum et al., 2020; O'Shea et al., 2017). The annual rise in the prevalence of SCI is so alarming that it cannot be overlooked and has been declared a public health problem (Cofano et al., 2019). SCI can lead to disability, paralysis and neural dysfunction, causing huge physical and mental damage to patients (Cofano et al., 2019; O’Shea et al., 2017). In general, SCI can be classified into primary SCI and secondary SCI (Anjum et al., 2020). In recent years, with in-depth research on the pathological mechanism of SCI, a series of repair strategies are employed to treat SCI, including repressing inflammatory response, restoring short-distance neural connectivity and function, regulating circuit reorganization in spared neural tissue and neurodegenerative strategies (Russo and McGavern, 2016; DeBrot and Yao, 2018). Among these, neurodegenerative strategies are an effective to treat SCI.

The neurodegenerative strategy is a treatment technique that replaces damaged cells and axons through stimulating the endogenous repair mechanism or through cell transplantation (Wu et al., 2020). With the rapid development of molecular biology and cell therapy, stem cell (SC) therapy had gradually become a promising method to treat neurological diseases because it could replace damaged cells and synthesize both neurotrophic factors and molecules that stimulate neuro regeneration (Galieva et al., 2017). In 2006, induced pluripotent stem cells (iPSCs) were firstly reported by Shiya Yamanaka, which were generated by retroviral introducing four transcription factors (Klf4, Oct3/4, Sox2, and c-Myc) into mouse fibroblasts and parallel to embryonic stem cells in morphology, cell multiplication ability, cell differentiation ability, epigenetic modification status, and gene and protein expression (Takahashi and Yamanaka, 2006). Accumulating studies have revealed that iPSCs have significant value in new drug screening, cell replacement therapy, and the treatment of neurological diseases and cardiovascular diseases (Hockemeyer and Jaenisch, 2016; Kumar et al., 2018). In the treatment of SCI, studies have shown that iPSCs-derived cells such as neural crest cells, oligodendrocytes, and mesenchymal stromal cells can be safely transplanted into the mice model of SCI. These results indicated that these derived cells could integrate and differentiate into the desired phenotype, and promote functional recovery (Nagoshi and Okano, 2017; Csobonyeiova et al., 2019). More importantly, the generation of iPSCs could avoid the ethical and moral concerns caused by other stem cells (Jung et al., 2017; Kalluri and LeBleu, 2020). In a word, iPSCs have become a new therapeutic method for the treatment of SCI.

Exosomes are nano-sized vesicles with 30–150 nm in diameter that participate in intercellular communication (Pegtel and Gould, 2019). They can transport proteins and functional RNAs to perform long-distance cell signal transduction in cells or tissues (Kalluri and LeBleu, 2020). Recently, some researchers have indicated that iPSCs-Exo has been regarded as an effective treatment method for human heart diseases (Jung et al., 2017; Yang, 2018). iPSCs-Exo has many advantages including repairing tissue and lose cells and providing environmental support (Yang, 2018).

Generally speaking, exosomes possess biological functions *via* the luminal cargo, such as mRNAs, microRNAs (miRNAs), and proteins. MiRNAs, small non-coding RNA with length oscillating 22–25 nucleotides, are common exosomal components and impact a significant role in target cells or tissues (Ailawadi et al., 2015). MiRNAs are revealed to affect downstream gene expression at a post-transcriptional level (Winter et al., 2009). Reduced miR-199b-5p expression is observed in SCI (Zhou et al., 2016), however, whether miR-199b-5p exerts key functions in SCI *via* iPSCs-Exo is rarely reported.

Herein, we applied an SCI model *in vivo* and bone marrow-derived macrophage *in vitro* to study the treatment effects of iPSCs-Exo on spinal cord injury and the role of miR-199b-5p in the above treatment process. A schematic diagram of the study idea is shown in Graphical abstract.

## Materials and methods

### Animals

The experiments involving animals were approved by the Ethics Committee of Capital Medical University. Eight-week-old C57BL/6 mice (Laboratory Animal Center of Nanjing University, Nanjing, China) were housed under SPF conditions with enough food and water.

### Cell transfection

Murine embryonic fibroblasts (MEFs, ATCC) were cultured in FM medium (DMEM-Glutamaxl containing 1% penicillin/streptomycin and 10% FBS, Sigma-Aldrich, MO, United States) (Pajer et al., 2015). MEFs at passage one were induced as iPSCs which were incubated with an embryonic stem medium (ESC medium, Sigma-Aldrich). Mouse bone marrow-derived macrophage (BMDM) was maintained in DMEM medium (1% penicillin/streptomycin, 10% FBS, and 50 ng/mL macrophage-stimulating factor (MCSF), Sigma-Aldrich) (Pegtel and Gould, 2019). BMDM were treated with 50 ng/mL LPS and iPSCs-Exo for 12 h. BMDM in the blank group was incubated with LPS. The BMDM phenotypes were detected by RT-qPCR and immunofluorescence assays. Using Lipofectamine 2000 (Thermofisher, United States), the LPS-treated BMDM (1 × 10^5^ cells/well) were transfected with pcDNA-NC, pcDNA-Hgf, miR-199b-5p mimics, and mimics-NC (Shanghai GenePharma Co., Ltd.) for 48 h at 37°C.

### Induced pluripotent stem cells generation

MEFs (1 × 10^5^ cells/well) were plated into a 6-well plate which was coated with gelatin in Farrell’s medium (FM) (Sigma-Aldrich). After overnight, the medium was replaced with embryonic stem cells (ESC) medium with 1 mM valproic acid and 8 μg/mL purified cocktail (Oct4, Klf4, Sox2, and c-Myc). After incubation for 12 h, the medium was replaced with the normal ESC medium for 36 h. Meanwhile, the cocktail was added every two days. On the ninth day, cells were cultured in 10 cm dishes until ESC-like colonies appeared (iPSCs formed).

### Isolation and characterization of induced pluripotent stem cells-derived exosomes

Using Exosomes Isolation Reagent (Invitrogen, CA), exosomes were isolated from iPSCs. To acquire exosomes, the iPSCs were cultured in exosome-free media for 48 h. After centrifugation at 3,000 rpm for 15 min, the supernatants were transferred to a fresh tube, incubated with exosome-isolated reagent overnight, and then centrifuged at 15,000 rpm for 1 h to pellet exosomes. The pellet exosomes were resuspended with incomplete ESC medium or PBS. Transmission electron microscope (TEM) (FEI, United States) and nanoparticle tracking analysis (NTA) (ZetaView PMX110, Particle Matrix, Meerbusch, Germany) were used to detect the morphology and size of exosomes, respectively.

### Quantitative RT-PCR assay

Extraction of total RNAs was conducted using TRIzol reagent (ThermoFisher, United States). After reverse transcription, gene expression was determined using RT-qPCR assay with SYBR Green PCR Master Mix (Takara). Related gene expression was normalized to GADPH and U6 and calculated by the 2^−ΔΔCT^ method (Shams et al., 2022).

### Western blot assay

Total proteins were extracted from cells and exosomes by lysed in RIPA buffer (Beyotime, Shanghai, China). After being separated by SDS-PAGE, the protein bands were transferred onto PVDF membranes. Primary antibodies (anti-CD81 (ab155760), 1:1,000; anti-CD9 (ab92726), 1:1,000; anti-CD63 (ab59479), 1:1,000; anti-pan-AKT (ab8805), 1:2000; anti-AKT (ab38449), 1:2000; anti-GSK3β (ab32391), 1:2000; anti-pan-GSK3β (ab75814), 1:2000; anti-CREB (ab32515), 1:2000; and anti-pan-CREB (ab32096), 1:2000; anti-β-actin (ab8227), 1:2000) were used to incubate the membranes overnight at 4°C. After further incubating secondary antibodies for 1 h at 37°C, the Bio-Rad Image Lab was employed to visualize the protein bands (Ul Hassan et al., 2021).

### Animal model of spinal cord injury

Mice were subjected to pentobarbital anesthetization and underwent laminectomy. After clamping the transverse processes of T11 and T12, a weight drop injury at the exposed spinal cord dorsal surface was created using a 5 g rod dropped at a height of 5 cm. After closing the muscles and skin in layers, mice were housed in a controlled room of temperature and humidity. A schematic of the spinal cord injury process as shown in Supplementary Figure S1B was drawn using Figdraw. Once the SCI model was established (the successful model was demonstrated by Supplementary Video S1), mice were assigned into PBS and iPSCs-Exo groups that were injected with 200 μL PBS and iPSCs-Exo through the tail vein for 3 days, respectively. The recovery of spinal cord function was evaluated. The motor function assessment was evaluated by Basso, Beattie, Bresnahan (BBB) score as previously reported (Yu et al., 2019). After the experiment, mice were euthanized and their spinal cord tissue was analyzed.

### Bone marrow-derived macrophage immunofluorescence staining

The BMDM was permeabilizated with .1% triton. After blocking, BMDM was incubated with anti-iNOS (Abcam, ab283655, 1:1,000), anti-F4/80 (Abcam, ab16911, 1:1,000), anti-F4/80 (Abcam, ab16911, 1:2,000) and anti-Arg1 (Abcam, ab203490, 1:2,000) antibodies for 2 h. After being treated with secondary antibody for 1 h at 25°C, the DAPI solution was used for nuclear staining for 5 min. The images were observed using a confocal laser scanning microscopy (TCA SP8, Leica, Germany).

### Histological analysis

The transverse sections were produced on the injury epicenter of SCI. The morphology and injury site were detected through Mayer’s Haematoxylin and Eosin staining (H and E staining). These images were observed using a light microscope (BX53, Olympus Corporation).

### Cytokine assay

To detect pro-inflammatory (IFN-γ, IL-6, and G-CSF) and anti-inflammatory (IL-4) cytokines in the LPS-treated BMDM, the ELISA kit (Keygen, Nanjing, China) were utilized to measure the cytokine concentration.

### Dual-luciferase reporter gene assay

The Hgf-wild type (WT) and Hgf-mutated type (Mut) sequences were inserted into the pmiRGLO reporter vector (Synthgene Biotech, Nanjing, China). After co-transfection with miR-199b-5p mimics or mimic-NC, the luciferase activity of Hgf-WT or Hgf-Mut was detected using a dual-luciferase reporter assay kit (Promega) after 48 h of the transfections that were normalized with Renilla luciferase activity.

### Target gene prediction

The downstream target genes of miR-199b-5p were predicted through the TargetScan database, and the target genes were enriched and analyzed through the Metascape database. The binding sites of miRNA-mRNA were predicted by the TargetScan database.

### Statistical assay

Data analysis was completed using SPSS 19.0. Data from three repeated experiments are displayed as mean ± standard deviation (SD). A two-way repeated ANOVA was employed to compare BBB scores between groups over time. Other data analyzed were examined by Student’s t-test. Statistically significant results were obtained when two-sided *p* < .05.

## Results

### Isolation and identification of induced pluripotent stem cells-Exo

**FIGURE 1:**
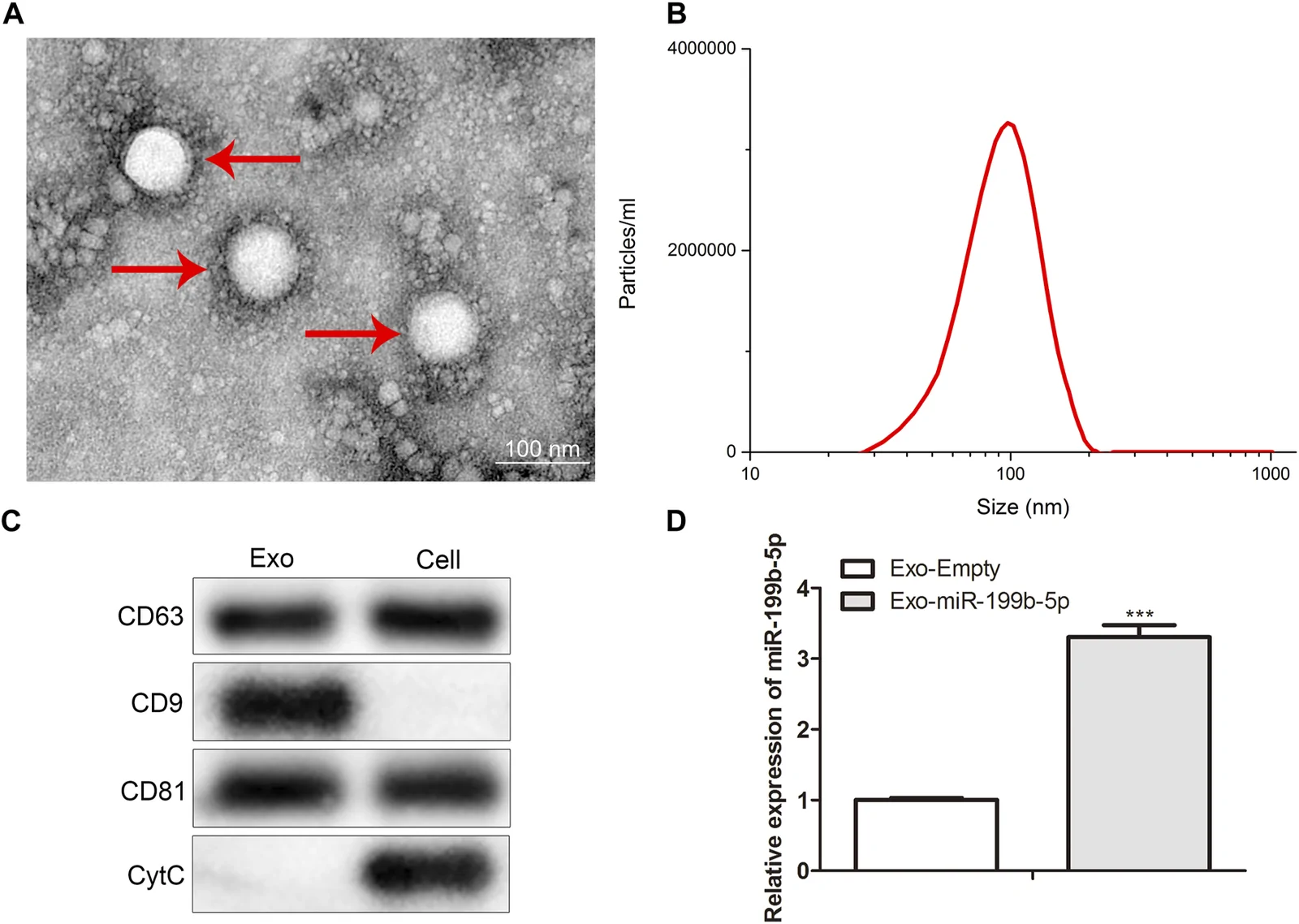
iPSCs-Exo was successfully isolated. **(A)** TEM displayed the morphology of iPSCs-Exo. **(B)** NTA revealed the concentration particles of iPSCs-Exo. **(C)** Western blot assay detected the protein levels of CD63, CD9, CD81 and CytC. **(D)** RT-qPCR detected the miR-199b-5p expression in isolated exosomes. ^***^ *p* < .001 vs. the Exo-Empty.

We first detected the morphology and size of the isolated iPSCs-Exo by using TEM and NTA. The spherical vesicle-like structure (30–80 nm) was observed by TEM (Figure 1A). NTA confirmed that the average exosome size was 100 nm (Figure 1B). Additionally, the expression levels of the exosome marker protein (CD9, CD63, CD81) and cell marker protein (cytochrome c, CytC) in isolated particles and whole iPSC lysates were determined by RT-qPCR. The results showed high expression of CD9, CD63, and CD81 in surfaces of vesicles, but Cyt c expression was not detected (Figure 1C), confirming that iPSCs-Exo were successfully isolated. At last, to verify whether the isolated exosomes contained miR-199b-5p, expression analyses were carried out in iPSCs-Exo and control by RT-qPCR and we found upregulation in gene expression of miR-199b-5p (Figure 1D).

### Induced pluripotent stem cells-exo improved motor function in spinal cord injury mice

**FIGURE 2:**
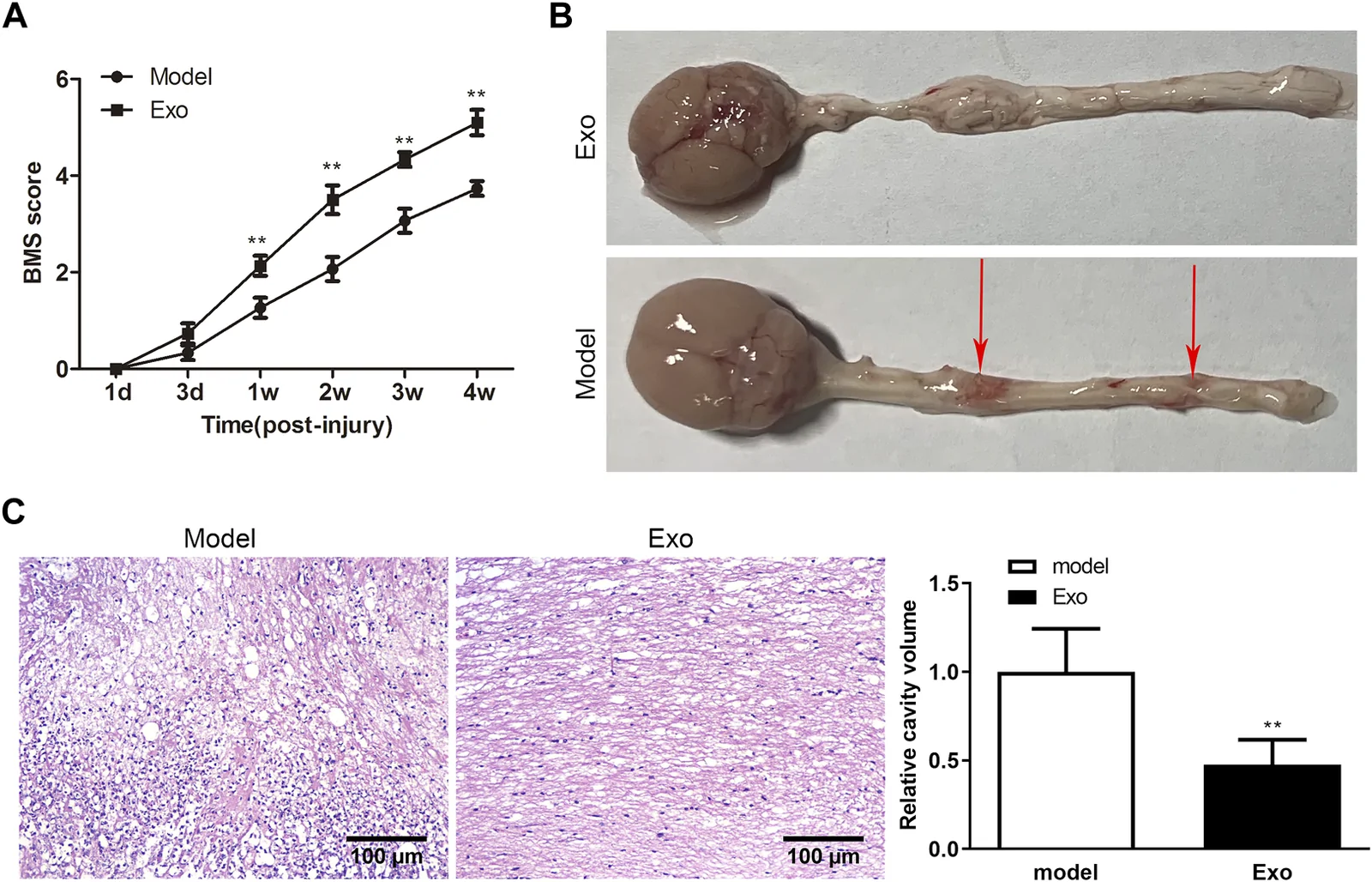
IPSCs-Exo improved motor function in SCI mice. **(A)** BMS scores. ^**^ *p* < .01 vs. the model group. **(B)** The overall shape of the spinal cord was observed in SCI model mice. The red arrow indicated the point of injured spinal cords in SCI model mice. **(C)** H&E staining of longitudinal SCI sections at 4 weeks. Scale bar = 100 μm.

To study the effects of iPSCs-Exo *in vivo*, SCI mice model was established. The BBB scores were calculated to evaluate the motor function of SCI mice after treatment with iPSCs-Exo. Meanwhile, the untreated SCI mice served as a control group. The BBB scores in mice injected with iPSCs-Exo increased in a time-dependent manner. At the 1st, 2nd, 3rd, and 4th weeks post-injection, the BBB scores of iPSCs-Exo groups were significantly higher than the control group (Figure 2A). Furthermore, to analyze the repair of injured spinal cords in SCI mice, the mice were sacrificed after 4 weeks of injury. The overall shape of the spinal cord demonstrated improvement in the iPSCs-Exo group when compared to the model group (Figure 2B). Meanwhile, histopathological analysis of the lesion area of SCI mice by H&E staining indicated that the cavity volume of mice in iPSCs-Exo groups was reduced when compared to the control (Figure 2C). This evidence suggested that iPSCs-Exo contributed to functional recovery after SCI.

### induced pluripotent stem cells-exo polarized macrophages to M2 phenotype *in vitro*

**FIGURE 3:**
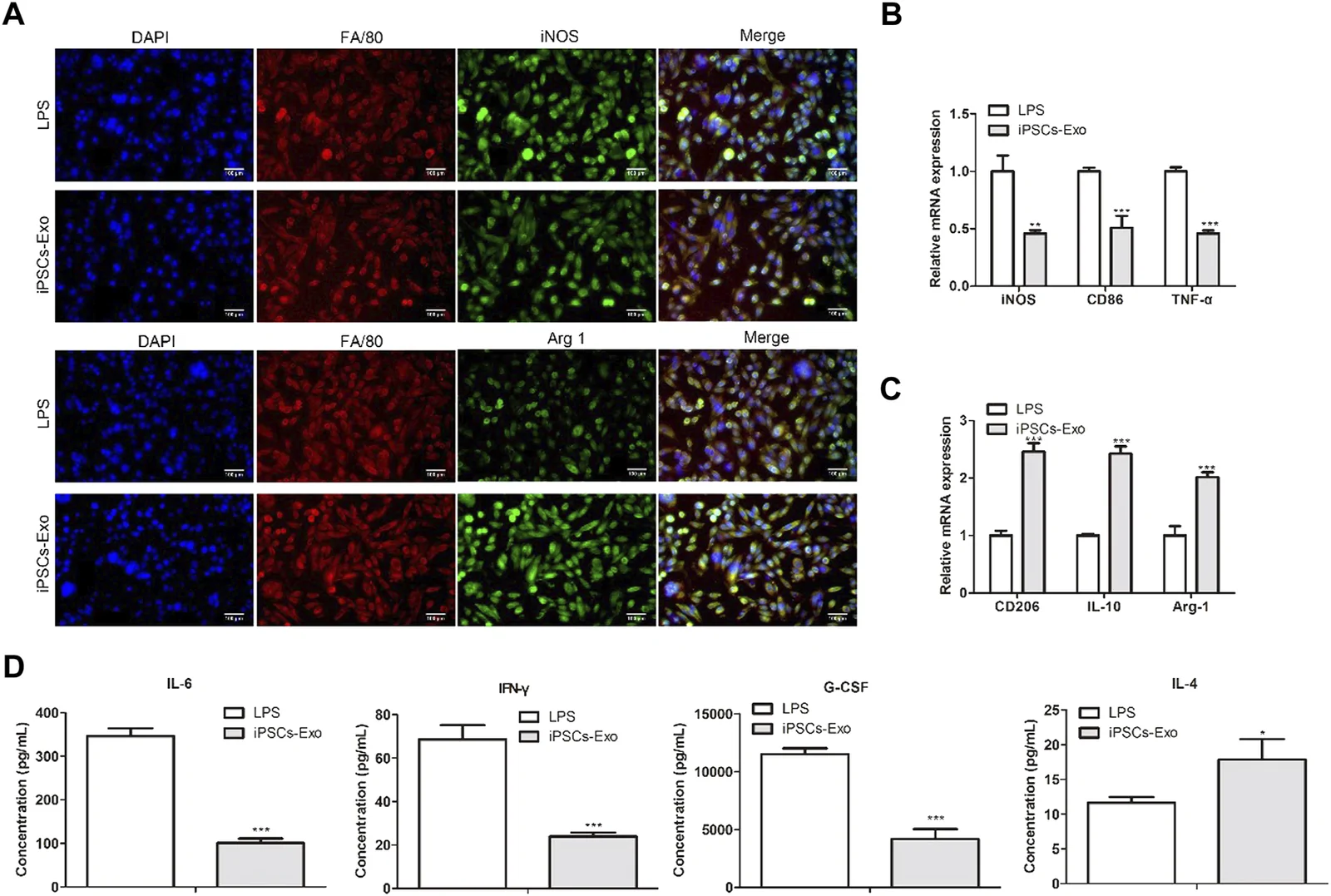
IPSCs-Exo polarized macrophages into M2 phenotype *in vitro*. **(A)** Immunofluorescence showed the expression of iNOS (M1 marker) and Agr1 (M2 marker). **(B)** RT-qPCR showed M1 marker expression (iNOS, CD86 and TNF-α). **(C)** RT-qPCR revealed M2 markers expression (CD206, IL-10 and Arg1). **(D)** ELISA detected the concentration of inflammatory factors (IL-4, IL-6, IFN-γ, and G-CSF). ^*^ *p* < .05, ^**^ *p* < .01, and ^***^ *p* < .001 vs. the control group.

Macrophages have been reported to mediate the inflammatory reaction *via* polarization into the M2 phenotype in SCI(Yang, 2018). However, the changes in macrophage function and cytokine secretion were still unclear after the macrophage was treated with iPSCs-Exo. Firstly, BMDM were treated with LPS (control group) and iPSCs-Exo, respectively. Compared to the LPS group, decreased iNOS (M1 marker) expression but increased Agr1 (M2 marker) expression was observed in the iPSCs-Exo group (Figure 3A). Moreover, RT-qPCR assay was employed to measure the expression levels of M1 and M2 macrophage phenotype markers. The results indicated that iPSCs-Exo reduced gene expression of M1 markers i.e., iNOS, CD86 and TNF-α while increasing M2 markers’ CD206, IL-10, and Arg1 expression (Figures 3B,C). Afterward, protein levels of IL-4, IL-6, IFN-γ, and G-CSF were quantified through ELISA in mice. We found increased production of IL-4 protein while IL-6, IFN-γ, and G-CSF protein levels were found to be decreased in the iPSCs-Exo mice group (Figure 3D). Overall, our results in Figure 3 demonstrated that iPSCs-Exo alters macrophage polarization and cytokine release in SCI.

### induced pluripotent stem cells-exo regulated spinal cord injury recovery through miR-199b-5p

**FIGURE 4:**
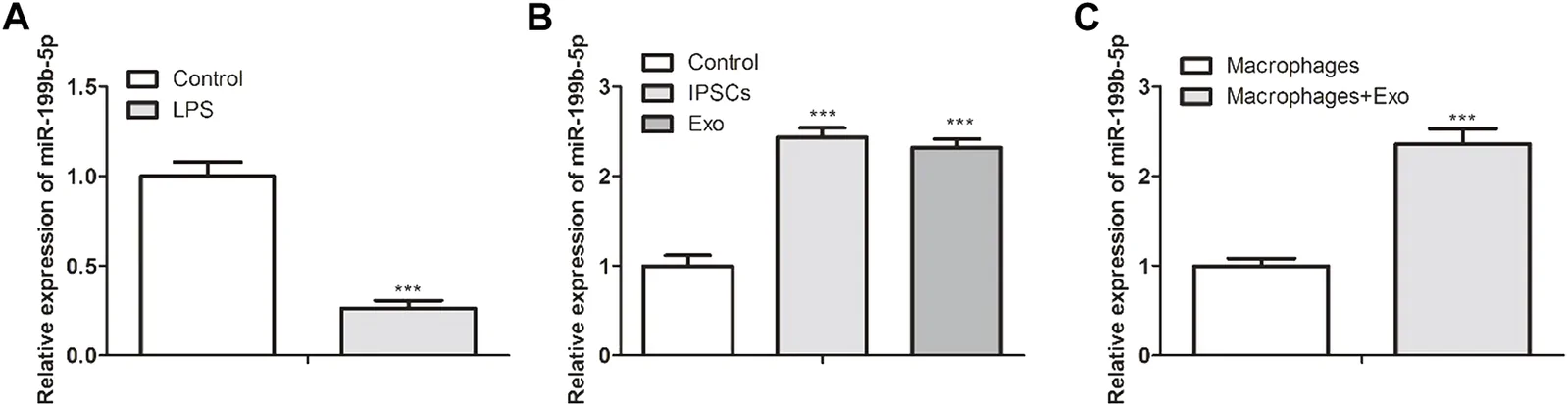
IPSCs-Exo regulated the spinal cord injury recovery through miR-199b-5p. **(A)** RT-qPCR showed miR-199b-5p expression in LPS-treated BMDM. **(B)** RT-qPCR revealed the miR-199b-5p expression in iPSCs and iPSCs-Exo. ^***^ *p* < .001 vs. the control group. **(C)** RT-qPCR revealed the miR-199b-5p expression in LBS-treated BMDM after treatment with iPSCs-Exo. ^***^ *p* < .001 vs. the macrophages group.

We further investigate whether iPSCs-Exo promoted macrophage polarization and SCI functional recovery *via* miR-199b-5p. Our qPCR results indicated decreased expression of miR-199b-5p in LPS-treated BMDM and increased expression in iPSCs and iPSCs-Exo (Figures 4A,B). Meanwhile, miR-199b-5p was enhanced expressed in LBS-treated BMDM after treatment with iPSCs-Exo (Figure 4C).

### MiR-199b-5p polarized macrophages to M2 phenotype and promoted neuronal regeneration *in vitro*

**FIGURE 5:**
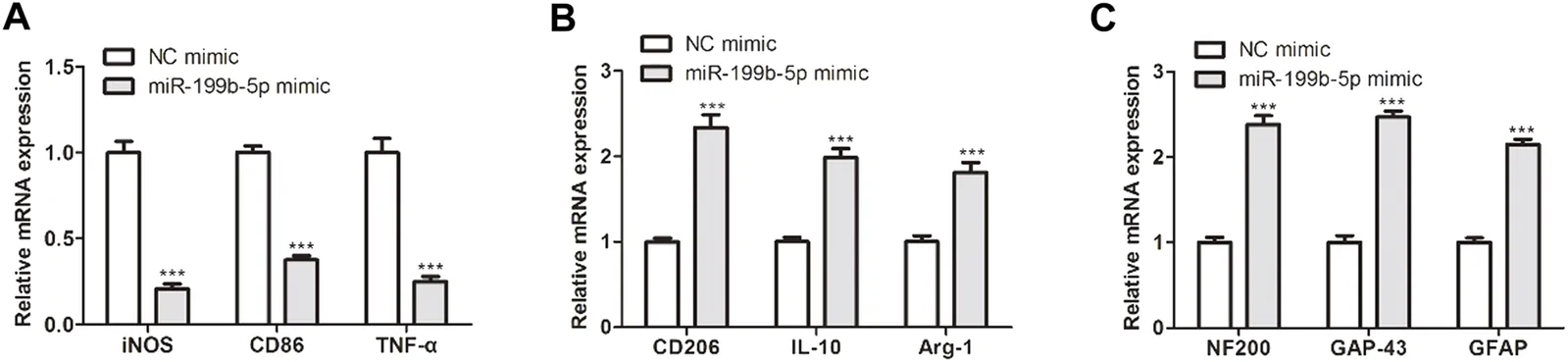
MiR-199b-5p polarized macrophages into M2 phenotype and promoted neuronal regeneration *in vitro*. **(A)** RT-qPCR detected the M1 marker expression (iNOS, CD86 and TNF-α) after transfection. **(B)** RT-qPCR showed the M2 marker expression (CD206, IL-10, and Arg1). **(C)** RT-qPCR measured the expression of NF200, GAP-43, and GFAPT. ^***^ *p* < .001 vs. the NC mimic.

To explore the function of miR-199b-5p in LPS-treated BMDM, miR-199b-5p was overexpressed in LPS-treated BMDM through transfection. We first detected the expression levels of M1 and M2 macrophage phenotype markers. As revealed in Figures 5A,B, miR-199b-5p overexpression downregulated iNOS, CD86, and TNF-α expression in M1 macrophage phenotype but upregulated CD206, IL-10 and Arg1 expression in M2 macrophage phenotype, suggesting that miR-199b-5p polarized macrophages from M1 to M2 phenotype *in vitro*. Moreover, NF200 and GAP-43 are the indicators of functional recovery of SCI while GFAP is an indicator of hindering the recovery of SCI (Yunna et al., 2020). The protein expression levels of NF200 and GAP-43 were increased while GFAP expression was decreased after the LPS-treated BMDM treated with miR-199b-5p mimics (Figure 5C), which suggested that miR-199b-5p overexpression might promote neuronal regeneration.

### Hgf was targeted by miR-199b-5p and hgf activated PI3K signaling pathway *in vitro*

**FIGURE 6:**
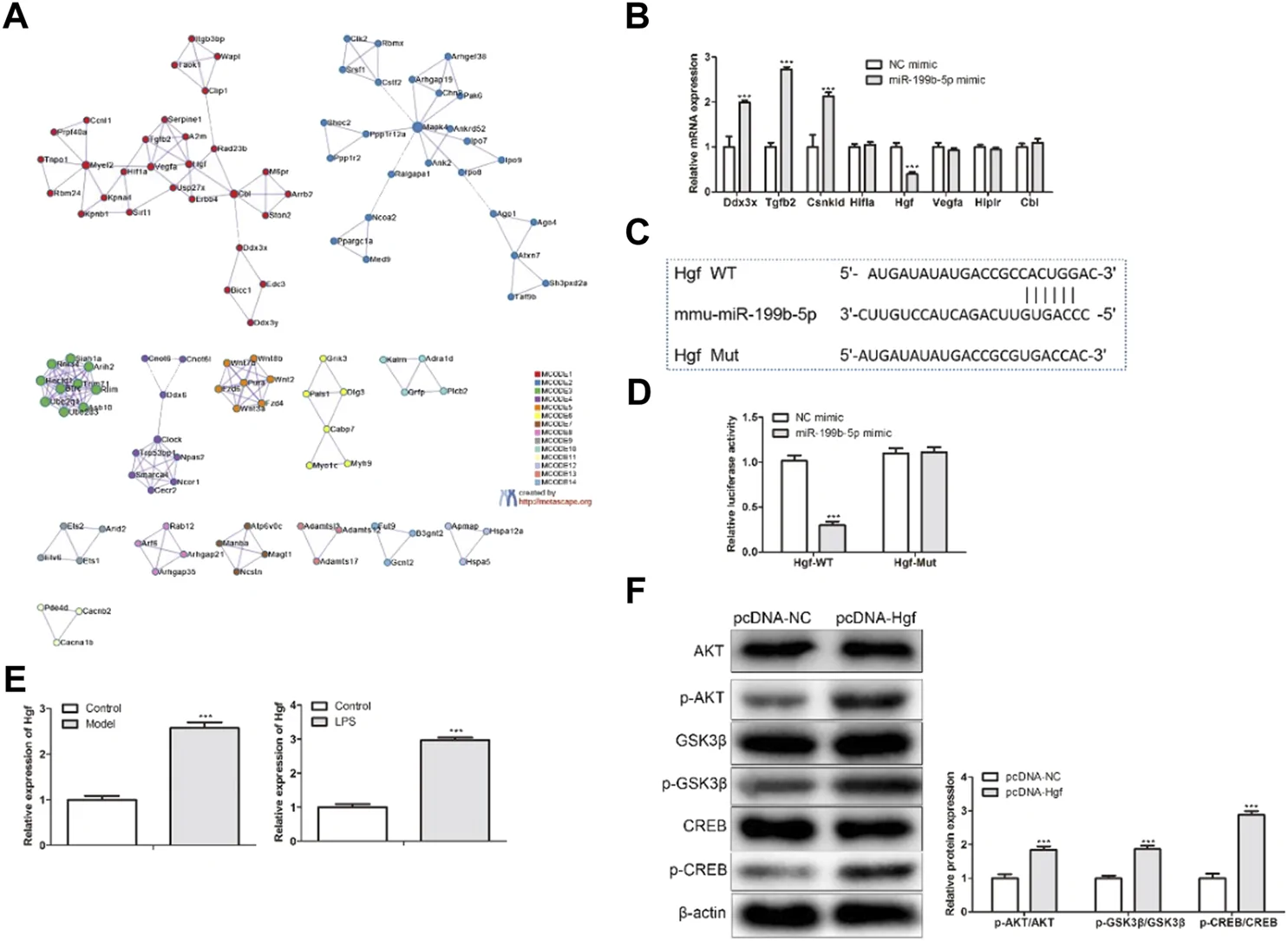
Hgf was targeted by miR-199b-5p and Hgf activated PI3K signaling pathway *in vitro*. **(A)** The TargetScan and Metasacpe databases predicted the target gene of miR-199b-5p. **(B)** RT-qPCR showed the expression of eight potential targets. **(C)** The predicted binding sequences of miR-199b-5p and Hgf by TargetScan. **(D)** The relative luciferase activity. ^***^ *p* < .001 vs. the NC mimic. **(E)** RT-qPCR showed Hgf expression in both the SCI mice model and LPS-treated BMDM. ^***^ *p* < .001 vs. the control group. **(F)** Western blot assay detected PI3K signaling pathway-related protein expression. The phosphor-protein expression was normalized with *β*-actin ^***^ *p* < .001 vs. pcDNA-NC.

To study the molecular mechanism of miR-199b-5p in macrophage polarization, the bioinformatics analysis (mirdbH and Metasacpe) was employed. Through this assay, eight potential targets (Ddx3x, Tgfb2, Csnk1d, Hifla, Hgf, Vegfa, Hip1r, and Cbl) were screened (Figure 6A). In addition, these potential targets are involved in many pathways (IRF5, ERK, JAK2, IL-4, Notch1, PI3K, et al.) to regulate macrophage polarization. Among them, PI3K signaling pathways were closely related (Figure 6A). Then, we measured the expression of eight potential targets after the LPS-treated BMDM treated with miR-199b-5p mimics. Hgf expression was lower than other targets’ expression (Figure 6B). Also, the putative binding site of miR-199b-5p was contained in the Hgf 3′-UTR (Figure 6C). Luciferase reporter assay indicated that only the luciferase activity of Hgf-WT was reduced with miR-199b-5p overexpression (Figure 6D). Subsequently, we detected the Hgf expression in the SCI mice model and LPS-treated BMDM. Hgf expression was enhanced both in the SCI mice model and LPS-treated BMDM (Figure 6E). More importantly, to explore the relationship between Hgf and the PI3K signaling pathway, the related proteins’ expression of the PI3K signaling pathway was measured after Hgf overexpression. The phosphorylated proteins (p-AKT, p-GSK3β, and p-CREB) in PI3K signaling pathway were increased expressed after the LPS-treated BMDM were treated with pcDNA-Hgf. These results suggested that Hgf overexpression could activate the PI3K signaling pathway (Figure 6F). In summary, Hgf was a target of miR-199b-5p, and Hgf overexpression could activate the PI3K signaling pathway.

### MiR-199b-5p promoted polarization from M1 macrophages to M2 phenotype through regulating hgf and PI3K signaling pathway

**FIGURE 7:**
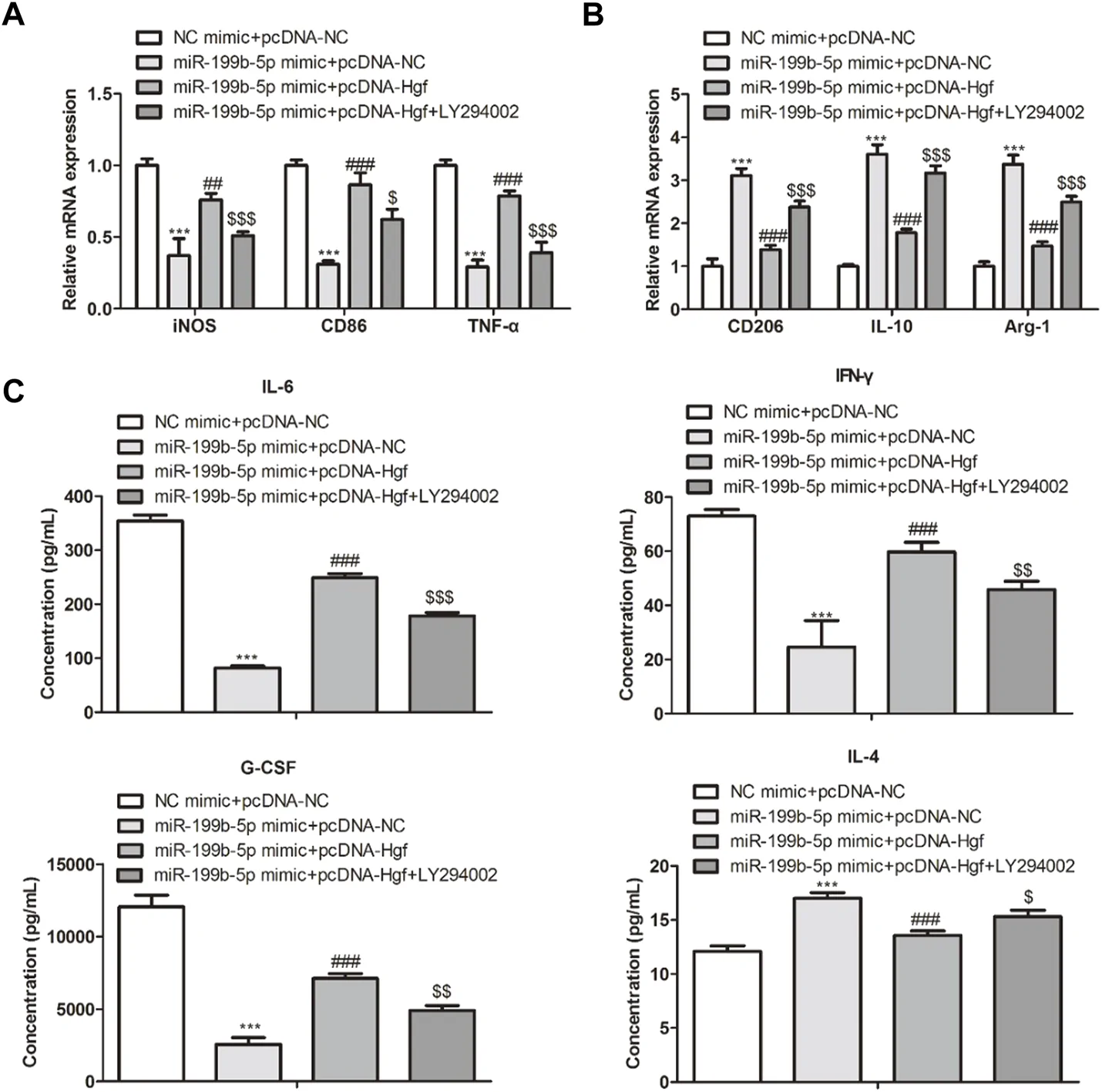
MiR-199b-5p regulated macrophage polarization *via* regulating Hgf and PI3K signaling pathways. **(A)** RT-qPCR showed M1 marker expression (iNOS, CD86 and TNF-α). **(B)** RT-qPCR revealed M2 marker expression (CD206, IL-10, and Arg1). **(C)** ELISA showed the concentration of IL-4, IL-6, IFN-γ, and G-CSF. ^***^ *p* < .001, ^##^ *p* < .01, ^###^ *p* < .001, ^$^ *p* < .05, ^$$^ *p* < .01, ^$$$^ *p* < .001 vs. NC mimic + pcDNA-NC.

To further verify if Hgf mediated the promotion of LPS-treated BMDM polarization was caused by miR-199b-5p, the pcDNA-NC, pcDNA-Hgf, and PI3K signaling pathway, inhibitor LY294002 were transfected into LPS-treated BMDM treated with miR-199b-5p mimics. It was observed that Hgf overexpression increased the expression of iNOS, CD86, and TNF-α in the M1 macrophage phenotype while decreasing the CD206, IL-10, and Arg1 expression in M2 macrophage phenotype (Figures 7A,B). These results were contrary to the results of miR-199b-5p mimics or LYP294002 on M1 and M2 marker expression. In addition, miR-199b-5p overexpression upregulated IL-4 but downregulate IL-6, IFN-γ, and G-CSF. The result was consistent with the result of LYP294002. However, the Hgf overexpression increased IL-6, IFN-γ, and G-CSF concentration while decreasing the expression of IL-4 (Figure 7C). Collectively data in this figure indicated that miR-199b-5p promoted macrophage polarization from M1 to M2 phenotype through regulating Hgf and PI3K signaling pathway. (Kalluri and LeBleu, 2020).

## Discussion

Over the past few decades, SCI has become a public health problem owing to its high morbidity and mortality and high medical cost and gradually become a hot spot (Cofano et al., 2019; McDonald and Sadowsky, 2002; O'Shea et al., 2017). With the rapid development of iPSCs therapy, it has gradually become an effective method to treat heart diseases and neurodegenerative diseases (Takahashi and Yamanaka, 2006; Wang et al., 2017). Accumulating studies have indicated that iPSCs are widely used to treat myocardial infarction, cardiac injury and dysfunction, spinal cord injury, and neurodegenerative diseases (Hockemeyer and Jaenisch, 2016; Kumar et al., 2018). IPSCs can not only repair damaged cells or neurons but also promote tissue regeneration (Khazaei et al., 2014). Therefore, iPSCs have provided a new opportunity for the treatment of SCI.

Exosomes, nano-sized vesicles, are implicated in the cell signal process by transporting the luminal cargos, like proteins and functional RNAs(Pegtel and Gould, 2019; Kalluri and LeBleu, 2020). Recently, several studies have verified that iPSCs-Exo has contributed to the treatment of heart diseases and SCI(Pajer et al., 2015; Zhang et al., 2022). Herein, we prepared the iPSCs-Exo with a mean particle size of 100 nm. In the SCI mice model, the function has been recovered and the inflammatory reaction has been regulated in iPSCs-Exo treated SCI, which indicated macrophages played a vital role in SCI pathological development. Then, we further explored the underlying mechanism in the following studies *in vitro*.

However, whether the iPSCs-Exo were related to macrophage polarization in SCI pathological process was still obscure. To explore the relationship between iPSCs-Exo and macrophage polarization, the LPS-treated BMDM were treated with iPSCs-Exo. The results revealed that iPSCs-Exo facilitated the polarization from M1 macrophage to M2 phenotype. Moreover, iPSCs-Exo increased IL4 expression but decreased IL-6, IFN-γ, and G-CSF expression. The above results indicated that the polarization fromM1 macrophage to M2 phenotype could be triggered by iPSCs-Exo *via* regulating the release of related cytokines, thus promoting the functional recovery of SCI. Nevertheless, how iPSCs-Exo regulate macrophage polarization and the SCI process needs further study.

Currently, MSCs-Exo is found to exert biological function *via* the regulation of miRNAs at the genetic level (Chen et al., 2018; Ajmal et al., 2022). For example, exosomal miR-146a-5p could alleviate the neuroinflammatory response in the development of ischemic stroke (Zhang et al., 2021). MiR-199b-5p is reported to be reduced expressed in acute SCI and microglia (Gao et al., 2019). In our study, we found that iPSCs-Exo regulated the SCI recovery *via* miR-199b-5p, which might be attributed to high miR-199b-5p expression in iPSCs-Exo. In addition, miR-199b-5p overexpression shifted the macrophage polarization *in vitro*. NF200, GAP-43, and GFAP were closely associated with neural regeneration. Therefore, these three protein expression levels could effectively reflect neuronal regeneration degree in SCI. When miR-199b-5p was overexpressed, NF200 and GAP-43 expression were increased and GFAP expression was decreased. Taken together, miR-199b-5p overexpression improved neural regeneration in SCI.

Hgf, a hepatotropic factor, is a tumor-related gene and participated in many diseases, such as tumors and the cardiovascular system (Nakamura and Mizuno, 2010; Yan et al., 2021; Klotz et al., 2021). For example, Hgf/MET pathway has become an effective target and biomarker in many cancers (Moosavi et al., 2019; Tao et al., 2020; Gao et al., 2021). In ischemic injury, Hgf and MET protect the heart by activating PI3K/Akt and MAPK signaling pathways (Gallo et al., 2015; Cai et al., 2022). In SCI, serval studies have proven that PI3K signaling pathway is related to macrophage polarization (Hua et al., 2007; Vergadi et al., 2017). Herein, we confirmed that Hgf was a target of miR-199b-5p. Meanwhile, Hgf expression was increased in the SCI mice model and LPS-treated BMDM, and Hgf overexpression activated the PI3K signaling pathway in LPS-treated BMDM. Furthermore, miR-199b-5p promoted polarization from M1 macrophages to M2 phenotype *via* regulating Hgf and PI3K signaling pathways.

## Conclusion

We demonstrated that iPSCs-derived exosomes (iPSCs-Exo) effectively improved motor function in SCI mice model *in vivo* and shifted the polarization from M1 macrophage to M2 phenotype and regulated related inflammatory factors expression to accelerate the SCI recovery in LPS-treated BMDM *in vitro*. Meanwhile, miR-199b-5p was a key player to modulate the effect of iPSCs-Exo in SCI. miR-199b-5p overexpression polarized macrophages into M2 phenotype and improved neural regeneration in SCI. More importantly, Hgf has confirmed a target of miR-199b-5p and Hgf overexpression activated the PI3K signaling pathway. Therefore, miR-199b-5p promoted macrophage polarization and SCI recovery by regulating Hgf and PI3K signaling pathways. The miR-199b-5p-bearing iPSCs-Exo might be an effective therapeutic target in the clinic. We will evaluate its clinical application value in future research.

## Data Availability

The original contributions presented in the study are included in the article/Supplementary Material, further inquiries can be directed to the corresponding authors.

## Supplementary material

SUPPLEMENTARY FIGURE S1
**(A)** Schematic diagram of research ideas and **(B)** the schematic diagram of the spinal cord injury process was drawn using Figdraw.

## Abbreviations

iPSCs-Exo, induced pluripotent stem cells-derived exosomes; SCI, spinal cord injury; MSCs, mesenchymal stem cells; miRNAs, MicroRNAs; MEFs, murine embryonic fibroblasts; MCSF, macrophage-stimulating factor; BMDM, bone marrow derived macrophage.
