# Correction: Induced pluripotent stem cells as natural biofactories for exosomes carrying miR-199b-5p in the treatment of spinal cord injury

**DOI:** 10.3389/fphar.2026.1953680

**Published:** 2026-08-19

**Authors:** Jun Li, Yingli Jing, Fan Bai, Ying Wu, Limiao Wang, Yitong Yan, Yunxiao Jia, Yan Yu, Benzhi Jia, Fawad Ali

**Affiliations:** 1 Department of Spinal and Neural Functional Reconstruction, China Rehabilitation Research Center, Beijing, China; 2 School of Rehabilitation Medicine, Capital Medical University, Beijing, China; 3 China Rehabilitation Science Institute, Beijing, China; 4 State Key Laboratory of Proteomics, National Center for Protein Sciences (Beijing), Beijing Institute of Lifeomics, Beijing, China; 5 College of Rehabilitation Medicine, Shandong University of Traditional Chinese Medicine, Jinan, Shandong, China; 6 Department of Spinal cord injury rehabilitation, Shanxi Kangfu Hospital, Xi’an, Shanxi, China; 7 Department of Pharmacy, Kohat University of Science and Technology, Kohat, Pakistan

**Keywords:** induced pluripotent stem cells-derived exosomes, spinal cord injury, microphage polarization, miR-199b-5p, Hgf, quantitative RT-PCR assay

The reference Xu, S., Tao, H., Cao, W., Cao, L., Lin, Y., Zhao, S.-M., et al. (2021). Ketogenic diets inhibit mitochondrial biogenesis and induce cardiac fibrosis. *Signal Transduct. Target. Ther.* 6, 54. doi: 10.1038/s41392-020-00411-4 was erroneously included in the article and cited in section **Discussion,** Paragraph 5. This reference has been replaced by Klotz, D. M., Link, T., Wimberger, P., and Kuhlmann, J. D. (2021). Prognostic relevance of longitudinal HGF levels in serum of patients with ovarian cancer. *Mol.Oncol.* 15(12), 3626–3638. doi: 10.1002/1878-0261.12949.

The reference Qu, Y.-Y., Zhao, R., Zhang, H.-L., Zhou, Q., Xu, F.-J., Zhang, X., et al. (2020). Inactivation of the AMPK-GATA3-ECHS1 pathway induces fatty acid synthesis that promotes clear cell renal cell carcinoma growth. *Cancer Res.* 80, 319–333. doi: 10.1158/0008-5472.CAN-19-1023 was erroneously included in the article and cited in section **Discussion**, Paragraph 5. This reference has been replaced by Yan, L., He, X., Tang, Y., Zhao, X., Luo, G., and Wang, X. (2021). HGF can reduce accumulation of inflammation and regulate glucose homeostasis in T2D mice. *J Physiol Biochem.* 77(4), 613–624. doi: 10.1007/s13105-021-00828-7.

The original version of this article has been updated.

