# Supplementary figures and images for: Induced pluripotent stem cells as natural biofactories for exosomes carrying miR-199b-5p in the treatment of spinal cord injury
Source: Front Pharmacol. 2023 Jan 10;13:1078761. doi: 10.3389/fphar.2022.1078761 (PMC9871459; doi:10.3389/fphar.2022.1078761)

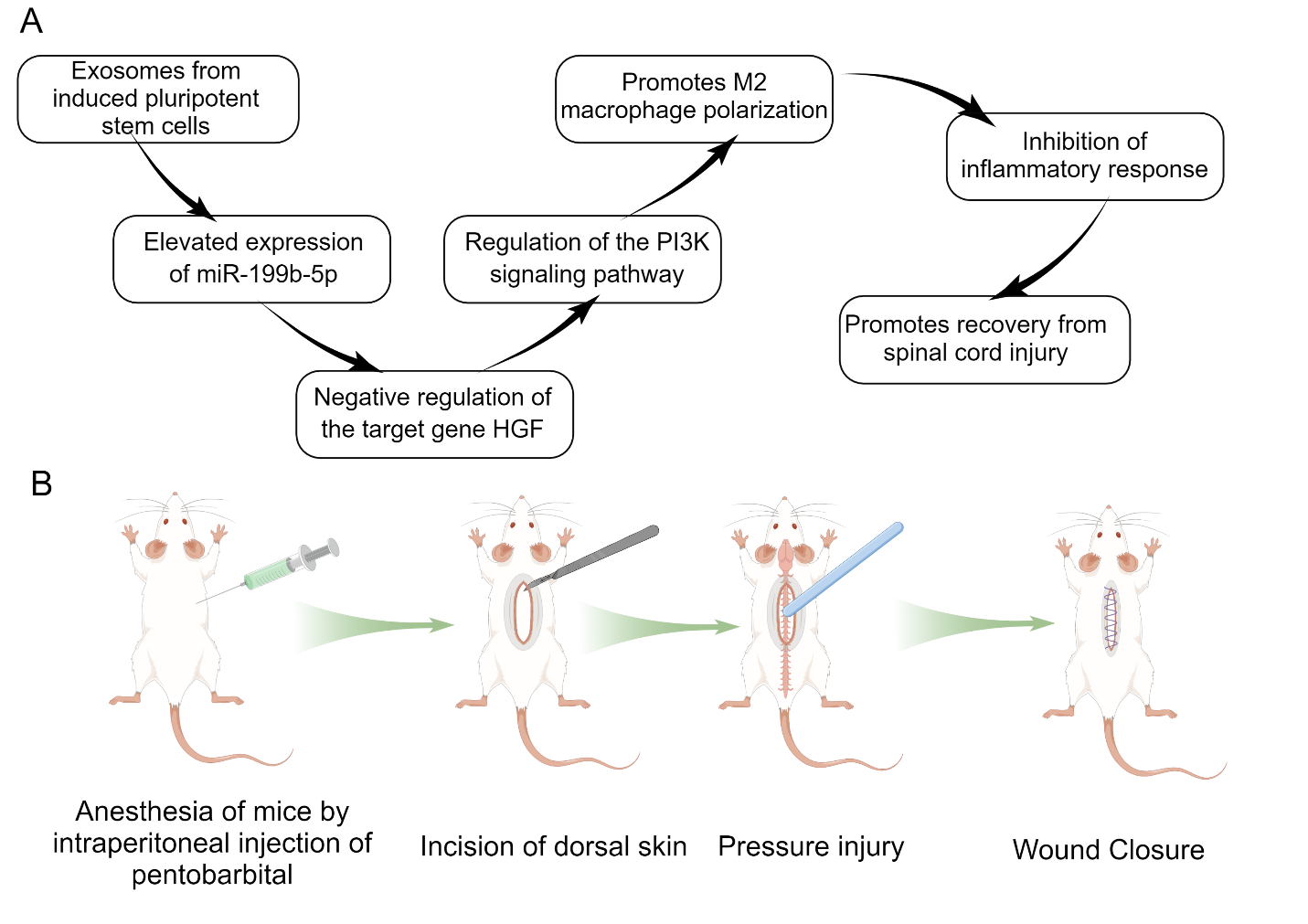

Supplement: Supplementary file 1 [file DataSheet1.docx]
